# Is There a Pediatric Role for the General Thoracic Surgeon in a General Hospital? A 22-Year Single-Center Experience

**DOI:** 10.3390/jcm13237231

**Published:** 2024-11-28

**Authors:** Francesco Petrella, Sara Degiovanni, Federico Raveglia, Andrea Cara, Enrico Mario Cassina, Francesca Graziano, Lidia Libretti, Emanuele Pirondini, Sara Vaquer, Antonio Tuoro, Giuseppe Foti, Alessandra Moretto, Alessandro Cattoni, Andrea Biondi, Adriana Balduzzi

**Affiliations:** 1 Department of Thoracic Surgery, Fondazione IRCCS San Gerardo Dei Tintori, 20900 Monza, Italy; sara.degiovanni@unimi.it (S.D.); federico.raveglia@irccs-sangerardo.it (F.R.); andrea.cara@irccs-sangerardo.it (A.C.); enricomario.cassina@irccs-sangerardo.it (E.M.C.); lidia.libretti@irccs-sangerardo.it (L.L.); emanuele.pirondini@irccs-sangerardo.it (E.P.); sara.vaquer@unimi.it (S.V.); antonio.tuoro@irccs-sangerardo.it (A.T.); 2 Biostatistics and Clinical Epidemiology, Fondazione IRCCS San Gerardo Dei Tintori, 20900 Monza, Italy; francesca.graziano@unimib.it; 3 Department of Medicine and Surgery, University of Milano-Bicocca, 20126 Monza, Italy; giuseppe.foti@unimib.it (G.F.); alessandro.cattoni@unimib.it (A.C.); andrea.biondi@unimib.it (A.B.); adriana.balduzzi@unimib.it (A.B.); 4 Department of Emergency and Intensive Care, Fondazione IRCCS San Gerardo Dei Tintori, 20900 Monza, Italy; alessandra.moretto@irccs-sangerardo.it; 5 Department of Pediatrics, Fondazione IRCCS San Gerardo Dei Tintori, 20900 Monza, Italy

**Keywords:** thoracic surgery, metapneumonic empyema, pleural effusion, pneumothorax, thoracic trauma

## Abstract

**Background**: Although general thoracic surgery is usually focused on adult patients, there are some settings of pediatric diseases which can benefit from thoracic surgical procedures. In this study, we retrospectively reviewed the contribution of general thoracic surgeons to pediatric patients in a high-volume hospital. **Methods**: From September 2002 to August 2024, 8897 consecutive patients were operated on; among them, 202 patients (2.2%) were younger than 18. Age, sex, operatory setting, side, indications, procedures, procedure duration, and perioperative mortality were collected for each patient. **Results**: Among the 202 patients younger than 18, 27 (13.3%) were 0–6 yo; 13 (6.4%) were 6–12 yo; 162 (80.1%) were 12–18 yo. In the first group, metapneumonic pleural effusion was the most frequent indication (44.4%) and chest drain the most frequent procedure (51.8%). No perioperative mortality was reported. In the second group, metapneumonic pleural effusion was the most frequent indication (30.7%) and chest drain the most frequent procedure (46.1%). No perioperative mortality was reported. In the third group, pneumothorax was the most frequent indication (41.3%) and bullectomy the most frequent procedure; (41.3%); one intraoperative death (0.4%) was reported in a case of major trauma. **Conclusions**: A general thoracic surgeon can effectively contribute to the surgical care of pediatric patients; in younger patients (<12 yo) urgent procedures related to infections are the most commonly performed; on the other hand, in patients aged between 12 and 18, elective procedures are more commonly performed, these being sympathectomy for hyperhidrosis and bullectomy for pneumothorax the most frequent.

## 1. Introduction

General thoracic surgery is usually focused on adult patients and treats both malignant and benign conditions. Thoracic malignancies—such as lung cancer, mesothelioma, thymoma, and esophageal cancer—represent the most common neoplastic diseases [1,2,3,4]; pneumothorax, infectious diseases, and thoracic trauma are the most frequent benign diseases—although not the only ones—which are treated by thoracic surgeons [5,6,7]. The above-mentioned diseases, in particular the oncologic ones, are rarely diagnosed among pediatric populations and this is the reason why general thoracic surgeons do not usually perform surgical procedures on pediatric patients; in addition, pediatric surgeons, subspecializing in thoracic diseases, actually treat several pathologic conditions such as congenital cystic adenomatoid malformations and other pediatric thoracic diseases, including major cardiothoracic malformation, which are managed together with congenital cardiac surgeons. In Italy, in most general hospitals, pediatric surgery specialists are not present and thoracic surgeons are asked to manage some conditions requiring thoracic surgical procedures in pediatric patients, usually in urgent or emergency settings. In this paper, we retrospectively reviewed our 22-year experience at a high-volume general hospital, a referral center for major trauma, with a high-volume pediatric department and a leading center for hematological pediatric diseases, despite not having a dedicated pediatric surgery division.

The role of thoracic surgery in pediatric patients is usually limited to just a few scenarios such as quite rare congenital malformations or metabolic diseases, some complications of infectious diseases potentially requiring surgical treatment, and major traumas, besides oncology. In several dedicated pediatric centers, thoracic surgical procedures are performed by pediatric surgeons, usually sub-specializing in pediatric thoracic diseases or cooperating with adult general thoracic surgeons or congenital cardiac surgeons. In many general hospitals, where pediatric surgery specialists are not present, adult general thoracic surgeons are asked to manage several clinical conditions, quite often in urgent or emergency settings. It is therefore important to provide general thoracic surgery residents with dedicated training in pediatric thoracic surgery, thus focusing on selected clinical indications and basic procedures; on the other hand, it is paramount for adult thoracic surgeons to acquire the proper skills to suggest a transfer to dedicated pediatric centers, after stabilizing the young patient, in more complex cases not amenable to safe treatment in centers without pediatric surgeons [8].

Childhood pneumonia is an important cause of morbidity and mortality and represents one of the most frequent reasons for pediatric hospital admissions [9,10]. Community-acquired pneumonia represents the first infectious cause of death in children younger than five years and about 12% of cases advance towards severe scenarios complicated by parapneumonic effusion and empyema [11]. The prevalence of empyema in pediatric patients is growing and nowadays it ranges from 0.6% to 2% of all community-acquired pneumonia; its mortality ranges from 1% in healthy patients to 40% in immunocompromised children. Antibiotics represent the first-line treatment for parapneumonic effusion and empyema; chest drainage, local fibrinolytic therapy, and video-assisted surgical debridement thoracotomy and decortications represent additional more aggressive options which might be required in the event of failure of standard antibiotic therapy [12]. Current clinical practice guidelines suggest adopting chest drainage procedures in addition to antibiotic therapy in patients presenting mild to large parapneumonic effusion and empyema; however, there is no standardized treatment and approaches vary according to the site of effusion and physician preference [13]. It is believed that effusions start as free-flowing fluid, then develop loculations and finally evolve into a purulent effusion consolidating into a fibrinous peel [14]. Limited effusions do not usually require a chest drain, while larger effusions with clinical impact on respiratory function and chest wall dynamics should be promptly evacuated to allow clinical improvement [15]. In our experience, meta-pneumonic pleural effusion was the most frequent indication to a surgical approach both in the 0–6 and 6–12 groups. A good command of flexible bronchoscopy procedures is very useful in this setting, in order to perform bronchoalveolar lavages and provide more detailed information about lung infections; on the other hand, rigid bronchoscopy is mainly indicated in the case of foreign body inhalation and should be performed in highly specialized centers by a dedicated team [16,17].

Pneumonia in children represents a serious cause of morbidity and mortality, being one of the most common reasons for hospital admissions in children [11]. Community-acquired pneumonia is the most frequent infectious cause of death in children younger than 5 years. About 12% of children affected by pneumonia evolve to critical disease and para-pneumonic effusions and empyema represent the most common and severe complications. The prevalence of empyema complicating childhood community-acquired pneumonia ranges from 0.6% to 2% of all diagnosed cases and rises to 28% to 53% among hospitalized children. Post-pneumonia empyema discloses a significant mortality rate which ranges from 1% in the healthy population to 40% in the immunocompromised patient [18]. Although many studies in the adult population have analyzed several different approaches to post-pneumonia effusion and empyema, the resulting findings show limited suitability to pediatric patients because adult patients usually present significant medical comorbidities and sepsis, which are responsible for sensible discrepancies both in mortality and total length of stay when compared to pediatric patients. The most effective therapeutic options both for post-pneumonia effusions and empyema are antibiotic therapy, pleural drainage alone, pleural drainage with fibrinolytics, surgical debridement or lung decortication by a video-assisted approach (VATS) or standard thoracotomy. Nowadays, chest drainage is recommended by current clinical practice guidelines, in addition to standard antibiotic therapy in case of mild or large para-pneumonia effusions and empyema, although there is no international consensus on the topic and indications vary according to surgeon experience, institution, and location [11]. Although rare, complications of more aggressive treatments with surgical drainage or debridement have been reported, like hemothorax, pneumothorax, and hemopneumothorax; some cases of hemothorax following fibrinolytic therapy have been reported as well as potential anesthetic risks when surgical procedures are required. According to recent meta-analytic studies, pleural drainage with fibrinolytic therapy, video-assisted as well as thoracotomic surgical approaches, provided a 5- or 6-day shorter total length of stay when compared to pleural drainage alone. In addition, no significant differences were observed in terms of total length of stay between VATS and thoracotomic approaches when compared to pleural drainage with fibrinolytic therapy. In terms of overall treatment costs, pleural drainage with fibrinolytic treatment was significantly cheaper when compared to the VATS approach. According to the literature results, long term prognosis of para-pneumonic effusions and empyema in children is usually favorable; at 1-year follow up, in fact, normal spirometric results and almost negative chest X-rays have been reported in 94% and 98% of patients, respectively [18]. In addition, all children were asymptomatic and their quality of life was defined as normal according to the Pediatric Quality of Life Inventory questionnaire [18]. The small number of children disclosing some spirometric anomalies at 1-year follow up disclosed only a moderate obstructive ventilatory deficit, which normalized over time regardless of the type of therapy adopted to treat para-pneumonia effusions and empyema. Pediatric mortality of para-pneumonia effusions and empyema was confirmed to be very low, ranging from 0% to 1% in several retrospective series [11], in clear contrast to adult mortality rate rising up to 20%, in most cases because of concurrent severe comorbidities and immunocompromised status. In the light of its favorable outcomes and limited costs, pleural drainage with fibrinolytic therapy might be considered as a reasonable first-line therapy of pediatric para-pneumonia effusions and empyema; on the contrary, a surgical approach should be limited to cases of failed conservative treatments. When the first-line therapy is represented by pleural drainage alone, fibrinolytic therapy should be promptly added whenever a rapid improvement is not observed, given that no clear contraindications have been reported and the favorable impact of fibrinolytic therapy on total length of stay is well known.

The objective of this study was to evaluate the possible contribution of general thoracic surgeons to pediatric patients, in a high-volume hospital, without a dedicated department of pediatric surgery.

## 2. Materials and Methods

### 2.1. Study Population

This was a single-center, retrospective, observational study conducted in accordance with the Declaration of Helsinki as revised in 2013 [19].

In this study, we enrolled consecutive patients who were operated on from September 2002 to August 2024 at the Division of Thoracic Surgery of the Fondazione IRCCS San Gerardo dei Tintori, Monza, Italy.

Inclusion criteria included age < 18 years. Pediatric patients were split into three groups depending on the age: 0–6 years (toddlers), 6–12 years (children), and 12–18 years (adolescents).

We recorded for each patient age, sex, operative setting, side, indications, procedures, procedure duration, number of procedures, and perioperative mortality.

Being a study focusing on procedures conducted in emergency as well as elective settings, written informed consent to undergo the procedure and for the use of clinical and imaging data for scientific or educational purposes, or both, was obtained when the patients’ parents were able to express their will in a timely manner.

Due to the pure retrospective nature of the study, the absence of any biological materials’ use, and the approved institutional data base adopted, approval by the medical ethics committee was waived.

### 2.2. Statistical Analysis

Descriptive characteristics were reported as frequency and percentage for categorical data, mean and standard deviation (sd) or median, and I-III quartile for continuous variables, where appropriate.

Comparison between descriptive characteristics among the three age categories was performed through the chi-squared or Kruskal–Wallis tests depending on the nature of the variable. All analyses were performed using RStudio (version 4.3.1). A two-tailed *p*-value of 0.05 was considered significant.

## 3. Results

From September 2002 to August 2024, out of the 8897 consecutive patients were operated on at the Division of Thoracic Surgery of the Fondazione IRCCS San Gerardo dei Tintori, 159 (1.8%) (2.2%) were younger than 18 with a mean age of 13.92 years old (standard deviation, sd = 4.74).

In the study population, 23 (14.5%) were 0–6 years (toddlers), 13 (8.2%) were 7–12 years (children), and 123 (77.4%) were 12–17 years (adolescent). A total of 51 (32.1%) were female and 108 (67.9%) male without statistical differences among the three age groups (*p* = 0.152). The male age group was 4.05 (sd = 1.34), 9.10 (sd = 1.61), and 16.28 (sd = 1.42), for 0–6 yo, 6–12 yo, and >12 yo, respectively.

Regarding the number of surgical operations, among the 159 patients, 118 underwent surgery only once, 39 underwent surgery twice, and two underwent surgery three times, for a total of 202 surgical operations. No intra- or in-hospital operative mortality was reported in toddlers and children; only one intraoperative death occurred in the adolescent group in a case of major trauma.

In detail, the 0–6 yo group (toddlers) included 23 patients and 27 interventions. As shown in Table 1, 18 (66.6%) were urgent and nine (33.4%) elective procedures; 10 (37%) on the right side, nine (33.3%) on the left side, and in the remaining eight (29.6%) cases side was not applicable. Indications were: metapneumonic pleural effusion, 12 (44.4%) cases; alveolar pneumopathy/NOS five (18.5%) cases; atelectasis, three (11.1%) cases; hemothorax, two (7.4%) cases; pneumothorax complicating lung abscess, two (7.4%) cases; metapneumonic empyema, two (7.4%) cases; mediastinal mass, one (3.7%) case. Procedures: chest drain, 14 (51.8%) cases; operative bronchoscopy, seven (25.9%) cases; decortication, three (11.1%) cases; mediastinal biopsy, one (3.7%) case; thoracocentesis, one (3.7%) case; wedge resection, one (3.7%) case. Procedure duration ranged from 5 to 140 min with a median time of 15 min and a mean time of 24 min. No intra- or in-hospital operative mortality was reported.

The 6–12 yo group consisted of 13 patients with one thoracic surgery each; nine (69.2%) urgent and four (30.8%) elective procedures; seven (53.8%) on the right side, four (30.7%) on the left side, and in the remaining two (15.3%) cases side was not applicable. Indications were: metapneumonic pleural effusion, four (30.7%) cases; recurrent pneumothorax, two (15.3%) cases; metapneumonic empyema, two (15.3%) cases; aspergilloma, one (7.6%) case; chest wall phlegmon, one (7.6%) case; alveolar pneumopathy/NOS, one (7.6%) case; pulmonary tuberculosis, one (7.6%) case; paraneoplastic effusion, one (7.6%) case. Procedures: chest drain, six (46.1%) cases; operative bronchoscopy, two (15.3%) cases; chest wall surgical biopsy, one (7.6%) case; bullectomy, one (7.6%) case; decortication, one (7.6%) case; lobectomy, one (7.6%) case; thoracocentesis, one (7.6%) case. Procedure duration ranged from 5 to 170 min with a median time of 15 min and a mean time of 35 min.

Finally, the 12–18 yo group included 123 patients and 162 thoracic surgeons; 41 urgent (25.3%) and 121 (74.7%) elective procedures; 67 (41.3%) on the right side, 74 (45.6%) on the left side, seven (4.3%) bilateral, and 14 (8.6%) not applicable. Indications were: pneumothorax, 67 (41.3%) cases; hyperhidrosis, 53 (32.7%) cases; oncohematological diseases, 15 (9.2%) cases; pectus excavatum, five cases (3%); trauma and hemothorax, four (2.4%) cases; foreign body inhalation, three cases (1.8%); other, 15 (9.2%) cases. Procedures: bullectomy, 67 cases (41.3%); sympathectomy, 53 (32.7%) cases; mediastinal biopsy and other biopsies of lymphatic structure, 15 (9.2%) cases; chest drain, eight (4.9%) cases; Nuss procedure, five (3%) cases; bronchoscopy procedures, four (2.4%) cases; thoracotomy, four (2.4%) cases; other procedures, six (3.7%) cases. Procedure duration ranged from 5 to 315 min with a median time of 35 min and a mean time of 46 min [Table 1 and Table 2].

## 4. Discussion

With this research, we try to address the potential role of a general thoracic surgeon in the diagnostic and therapeutic approach to pediatric thoracic diseases, in a setting without a dedicated pediatric surgical team. Although the vast majority of complex and congenital diseases should be properly treated by dedicated pediatric surgeons, general thoracic surgeons can effectively contribute to pediatric patients’ care, in particular in the emergency and infective settings.

Our hospital hosts a leading center for hematologic pediatric diseases and, therefore, the number of immunocompromised patients developing parapneumonic effusion is not negligible; for this reason, chest drainage and flexible bronchoscopy were the two most frequent procedures both in the 0–6 and 6–12 age groups.

Chest wall deformities, such as pectus excavatum, pectus carinatum, pectus arcuatum, and other more rare entities, represent a relatively frequent disease in pediatric patients. However, only five patients in the 12–18 group were treated at our center, which is not a referral center for this kind of pathology and this probably explains the limited number of treated patients [20].

Although very rare, pediatric thoracic trauma—not limited to chest drainage insertion and requiring a surgical approach—is a challenging scenario which thoracic surgeons might be asked to deal with in a complex emergency setting. We performed four emergency thoracotomies with one intraoperative death in a case of a patient presenting with an aortic rupture; this was the only intraoperative death (1/202) accounting for 0.4% of the entire population [21,22,23]. Although thoracic traumas are reported less frequently in children than adults, they represent the single most frequent cause of morbidity and mortality in children aged 1 to 14 years [24]. Thoracic injury represents only 5–12% of the admissions to trauma centers, but it may be related to greater lethality. Thoracic trauma alone in pediatric population carries a 5% mortality; however, it rises to 25% when abdominal or head lesions are superimposed. The diagnosis of head, chest, and abdominal injuries together may be fatal in nearly 40% of cases [24]. The greater elasticity of the rib cage in children allows the anterior ribs to be squeezed to reach the posterior ribs; as a consequence, lung contusion is more common while rib fracture occurs less commonly in pediatric patients than adult. After bony ribs calcification, rib and sternal fractures as well as flail chest occur more frequently; in the same way, as the bones are not fully ossified, children are more likely to suffer bony injuries without X-ray anomalies [24].

The population aged 12–18 disclosed many characteristics close to the adult population; it was the only group in which elective surgery (74.7%) was more frequent than urgent surgery (25.3%) while in the other groups urgent procedures were much more frequent, representing 66.6% in the 0–6 population and 69.2% in the 6–12 population. Similarly, the most frequent indications were pneumothorax (41.3%) and hyperhidrosis (32.7%) which represent the most frequent indications in young adults too. The pediatric conditions referred to the general thoracic surgeon depend on the emergency room accesses but may also be strongly associated with the elective nature of the routine activity in the pediatric department.

The pediatric department of our hospital has a strong commitment towards hematology, hematopoietic stem cell transplantation, and innovative cell therapy. T-lymphoblastic lymphoma would thus be the most frequent malignancy referred. In other settings, non-hemopoietic solid tumors could also be taken care of and be referred to the general thoracic surgeon, for diagnostics and therapeutical drainage.

This paper has several limitations: this is a retrospective paper with limited generalizability to other healthcare settings, due to the peculiar organization of our hospital where there is not a dedicated service of pediatric surgery. Moreover, our study covers a very long time span (22 years), thus making it possible to enroll a proper number of events in each subgroup of patients. On the other hand, this could affect the homogeneity of data, because protocols, surgeons, and management policies—in particular regarding trauma pathways—have changed over time. However, since the year 2000, the emergency and referral pathways for thoracic trauma have been almost uniform, thus permitting construction of a homogeneous data base.

As far as we know, this study focused on a peculiar hospital assessment where—although there is not a dedicated pediatric surgery department—general thoracic surgeons provide standard care for pediatric patients, in particular in cases of emergency and infective disease; this is the first report of this type and we did not find any previous similar experience in the recent literature.

We strongly recommend to thoracic surgeons in training a dedicated training period in pediatric departments to familiarize themselves with a specific population of patients with quite different needs from adult patients.

## 5. Conclusions

General thoracic surgeons can effectively contribute to pediatric surgical care in some settings, when no dedicated pediatric surgeons are available. Younger patients (0–12) usually require surgical therapy of metapneumonic pleural effusion or empyema in acute settings; on the other hand, older patients (12–18) are usually referred to general thoracic surgeons in elective settings for pneumothorax and hyperhidrosis, conditions frequently observed in young adults.

## Figures and Tables

**Table 1:** Clinical features of the study population (*n* = 159 patients), overall and among age groups.

	Overall	Group 1—Toddlers (0 < 6)	Group 2—Children (6 < 12)	Group 3— Adolescent (12 < 18)	*p*-Value
Patients *n* (%)	159	23	13	123	
Number of interventions (%)					
1	118 (74.2)	19 (82.6)	13 (100.0)	86 (69.9)	0.152
2	39 (24.5)	4 (17.4)	0 (0.0)	35 (28.5)	
3	2 (1.3)	0 (0.0)	0 (0.0)	2 (1.6)	
Sex (%)					
F	51 (32.1)	9 (39.1)	3 (23.1)	39 (31.7)	0.602
M	108 (67.9)	14 (60.9)	10 (76.9)	84 (68.3)	
age (mean (sd))	13.92 (4.74)	4.05 (1.34)	9.10 (1.61)	16.28 (1.42)	-

**Table 2:** Details of procedures.

	Overall	Group 1—Toddlers (0 < 6)	Group 2—Children (6 < 12)	Group 3—Adolescent (12 < 18)	*p*-Value
N of patients	159	23	13	123	
N of interventions (%)	202	27	13	162	
Operative setting					
elective	134 (66.3)	9 (33.3)	4 (30.8)	121 (74.7)	<0.001
urgent	68 (33.7)	18 (66.7)	9 (69.2)	41 (25.3)	
Side					
right	84 (42.0)	10 (37.0)	7 (63.6)	67 (41.4)	0.186
bilateral	9 (4.5)	2 (7.4)	0 (0.0)	7 (4.3)	
not applicable	20 (10.0)	6 (22.2)	0 (0.0)	14 (8.6)	
left	87 (43.5)	9 (33.3)	4 (36.4)	74 (45.7)	
Diagnosis by ICD-9-CM					
infectious diseases	33 (16.3)	17 (63.0)	11 (84.6)	5 (3.1)	<0.001
oncology	15 (7.4)	1 (3.7)	0 (0.0)	14 (8.6)	
trauma/foreign body inhalation	9 (4.5)	1 (3.7)	0 (0.0)	8 (4.9)	
hyperhidrosis	53 (26.2)	0 (0.0)	0 (0.0)	53 (32.7)	
pneumothorax	73 (36.1)	3 (11.1)	2 (15.4)	68 (42.0)	
other	19 (9.4)	5 (18.5)	0 (0.0)	14 (8.6)	
Diagnosis					
infectious diseases	33 (16.3)	19 (70.4)	9 (69.2)	5 (3.1)	<0.001
oncology	18 (8.9)	1 (3.7)	1 (7.7)	16 (9.9)	
trauma/foreign body inhalation	9 (4.5)	2 (7.4)	0 (0.0)	7 (4.3)	
hyperhidrosis	53 (26.2)	0 (0.0)	0 (0.0)	53 (32.7)	
pneumothorax	70 (34.7)	0 (0.0)	2 (15.4)	68 (42.0)	
other	19 (9.4)	5 (18.5)	1 (7.7)	13 (8.0)	
Type of procedure by ICD-9-CM (%)					
Sympathetic surgery	50 (24.8)	0 (0.0)	0 (0.0)	50 (30.9)	<0.001
Lung resection	65 (32.2)	1 (3.7)	2 (15.4)	62 (38.3)	
Chest wall surgery	10 (5.0)	0 (0.0)	1 (7.7)	9 (5.6)	
Diagnostic and operative endoscopy	13 (6.4)	7 (25.9)	2 (15.4)	4 (2.5)	
Chest drain	28 (13.9)	15 (55.6)	6 (46.2)	7 (4.3)	
Mediastinal/lymphatic biopsy	23 (11.4)	2 (7.4)	1 (7.7)	20 (12.3)	
Pneumothorax surgery	5 (2.5)	2 (7.4)	1 (7.7)	2 (1.2)	
Thoracotomy (explorative/emergency)	5 (2.5)	0 (0.0)	0 (0.0)	5 (3.1)	
Other	3 (1.5)	0 (0.0)	0 (0.0)	3 (1.9)	
Type of Procedure					
Sympathetic surgery	54 (26.7)	0 (0.0)	0 (0.0)	54 (33.3)	<0.001
Lung resection	7 (3.5)	1 (3.7)	1 (7.7)	5 (3.1)	
Chest wall surgery	11 (5.4)	0 (0.0)	1 (7.7)	10 (6.2)	
Diagnostic and operative endoscopy	13 (6.4)	7 (25.9)	2 (15.4)	4 (2.5)	
Chest drain	30 (14.9)	15 (55.6)	7 (53.8)	8 (4.9)	
Mediastinal/lymphatic biopsy	14 (6.9)	1 (3.7)	0 (0.0)	13 (8.0)	
Pneumothorax surgery	66 (32.7)	3 (11.1)	2 (15.4)	61 (37.7)	
Thoracotomy (explorative/emergency)	4 (2.0)	0 (0.0)	0 (0.0)	4 (2.5)	
Other	3 (1.5)	0 (0.0)	0 (0.0)	3 (1.9)	
Procedure duration (min)					
Mean (SD)	42.84 (42.62)	24.48 (28.28)	35.77 (48.30)	46.46 (43.48)	0.037
Median (I-III quartile)	32.50 (15, 50)	15 (10, 27.5)	15 (10, 25)	35 (25, 55)	<0.001

MPE: metapneumonic pleural effusion; AP: alveolar pneumopathy; NOS: not otherwise specified; ME: metapneumonic empyema.

## Data Availability

All data are available on request.
